# Prophylactic treatment of vestibular migraine^☆^

**DOI:** 10.1016/j.bjorl.2016.04.022

**Published:** 2016-06-02

**Authors:** Márcio Cavalcante Salmito, Juliana Antoniolli Duarte, Lígia Oliveira Golçalves Morganti, Priscila Valéria Caus Brandão, Bruno Higa Nakao, Thais Rodrigues Villa, Fernando Freitas Ganança

**Affiliations:** Universidade Federal de São Paulo (UNIFESP), Departamento de Otorrinolaringologia e Cirurgia de Cabeça e Pescoço, São Paulo, SP, Brazil

**Keywords:** Dizziness, Vertigo, Migraine, Prophylaxis, Treatment, Tontura, Vertigem, Enxaqueca, Profilaxia, Tratamento

## Abstract

**Introduction:**

Vestibular migraine (VM) is now accepted as a common cause of episodic vertigo. Treatment of VM involves two situations: the vestibular symptom attacks and the period between attacks. For the latter, some prophylaxis methods can be used. The current recommendation is to use the same prophylactic drugs used for migraines, including β-blockers, antidepressants and anticonvulsants. The recent diagnostic definition of vestibular migraine makes the number of studies on its treatment scarce.

**Objective:**

To evaluate the efficacy of prophylactic treatment used in patients from a VM outpatient clinic.

**Methods:**

Review of medical records from patients with VM according to the criteria of the Bárány Society/International Headache Society of 2012 criteria. The drugs used in the treatment and treatment response obtained through the visual analog scale (VAS) for dizziness and headache were assessed. The pre and post-treatment VAS scores were compared (the improvement was evaluated together and individually, per drug used). Associations with clinical subgroups of patients were also assessed.

**Results:**

Of the 88 assessed records, 47 were eligible. We included patients that met the diagnostic criteria for VM and excluded those whose medical records were illegible and those of patients with other disorders causing dizziness and/or headache that did not meet the 2012 criteria for VM. 80.9% of the patients showed improvement with prophylaxis (*p* < 0.001). Amitriptyline, Flunarizine, Propranolol and Topiramate improved vestibular symptoms (*p* < 0.001) and headache (*p* < 0.015). The four drugs were effective in a statistically significant manner. There was a positive statistical association between the time of vestibular symptoms and clinical improvement. There was no additional benefit in hypertensive patients who used antihypertensive drugs as prophylaxis or depressed patients who used antidepressants in relation to other prophylactic drugs. Drug association did not show statistically significant results in relation to the use of a single drug.

**Conclusions:**

Prophylactic medications used to treat VM improve the symptoms of this disease, but there is no statistically significant difference between the responses of prophylactic drugs. The time of vestibular symptom seems to increase the benefit with prophylactic treatment.

## Resumo

**Introdução:**

A migrânea vestibular (MV) é aceita atualmente como uma causa comum de vertigem episódica. O tratamento da MV envolve duas situações: as crises de sintomas vestibulares e o período intercrise. Para este último, pode-se utilizar algum método de profilaxia. A recomendação atual é que se utilizem os mesmos medicamentos profiláticos utilizados para a enxaqueca, o que inclui os β-bloqueadores, antidepressivos e anticonvulsivantes. A recente definição diagnóstica da migrânea vestibular torna escasso o número de estudos sobre seu tratamento.

**Objetivo:**

Avaliar a eficácia do tratamento profilático utilizado em pacientes em um ambulatório de MV.

**Método:**

Revisão de prontuários de pacientes com MV pelos critérios da *Bárány Society/International Headeache Society* de 2012. Foram pesquisados os medicamentos utilizados e resposta ao tratamento obtida através da escala visual-analógica (EVA) para tontura e cefaleia. Foram comparados os escores da EVA pré e pós-tratamento (a melhora foi avaliada em conjunto e individualmente por droga utilizada). Também foram pesquisadas relações com subgrupos clínicos dos pacientes.

**Resultados:**

De 88 prontuários estudados, 47 foram elegíveis. Incluiu-se os pacientes que preenchiam os critérios diagnósticos para MV, foram excluídos os prontuários ilegíveis e aqueles de pacientes com outro distúrbio causador de tontura e/ou cefaleia que não preenchiam critérios de 2012 para MV. Apresentaram melhora com a profilaxia 80,9% dos pacientes (*p* < 0,001). Amitriptilina, flunarizina, propranolol e topiramato apresentaram melhora para sintomas vestibulares (*p* < 0,001) e para cefaleia (*p* < 0,015). Os quatro medicamentos foram eficazes de forma estatisticamente significante. Houve relação estatística positiva entre tempo de sintoma vestibular e melhora clínica. Não houve benefício adicional para hipertensos que utilizaram anti-hipertensivos como profilaxia ou para os deprimidos que usaram antidepressivos em relação ao uso dos outros profiláticos. A associação de medicamentos não mostrou resultados estatisticamente significantes do uso de um medicamento isolado.

**Conclusões:**

Os medicamentos profiláticos utilizados para MV melhoram os sintomas dessa doença, porém não há diferença estatisticamente significante entre as respostas dos medicamentos profiláticos. O tempo de sintoma vestibular parece aumentar melhora obtida com o tratamento profilático.

## Introduction

Dizziness is one of the most common symptoms in medical practice, with an incidence of up to 30% a year and, despite its difficult approach, it is usually possible to reach a diagnosis.^1^, ^2^ Vestibular disorders are the main diseases that manifest with dizziness complaints and, among them, the most common are Benign Paroxysmal Positional Vertigo (BPPV), Vestibular Migraine (VM), Meniere's disease and vestibular neuritis, in decreasing order of frequency.^2^

Migraine is a multifactorial chronic disease. Its main symptom is headache, typically unilateral, pulsatile, associated with photophobia and phonophobia, nausea and vomiting.^3^, ^4^ The association between migraine headache and vertigo has been known for a long time and occurs three times more often than if it would by chance alone.^5^ In 1984, Kayan and Hood carried out a large study that showed a higher prevalence of otoneurological symptoms in patients with migraine, compared to patients with tension headache.^6^ Vestibular migraine as a specific entity, however, was only recently described in 1999 by Dieterich and Brandt,^7^ and is characterized by the association of vertigo episodes and migraine headache. To date, its definition is not uniform among the authors. Diagnostic criteria were proposed by Neuhauser in 2001^8^ and revised in 2012 by the Bárány Society and the International Headache Society (IHS),^9^ and was included in the third version of the International Classification of Headache Disorders – ICHD.^10^ The VM is now accepted as a frequent cause of episodic vertigo.

Treatment of VM involves two situations: treatment of attacks and prophylactic treatment. For the latter, some type of prophylaxis can be used.^11^, ^12^ The current recommendation is to use the same prophylactic drugs for VM as for migraine, which include β-blockers, antidepressants and anticonvulsants.^11^, ^12^, ^13^

The aim of this study was to evaluate the improvement in VM symptoms (headache and vestibular symptoms) after drug prophylaxis in patients with VM, treated at a VM clinic belonging to the Discipline of Otoneurology.

## Methods

An observational, longitudinal, retrospective study of medical record review was carried out. We evaluated the records of all patients treated at the Vestibular Migraine Outpatient Clinic of the Discipline of Otology and Neurotology, Department of Otolaryngology and Head and Neck Surgery of the Universidade Federal de São Paulo since its creation in February 2011 until June 2013. This study was approved by the Research Ethics Committee of the Universidade Federal de São Paulo (code 19615313.13.5.0000.5505).

The diagnostic criteria for vestibular migraine used were the criteria proposed in 2012 by the Bárány Society and the International Headache Society (IHD),^9^ included in the third version of the International Classification of Headache Disorders – ICHD.^10^ It included patients with “vestibular migraine” and “probable vestibular migraine.” As the Vestibular Migraine Outpatient Clinic of this Universidade Federal de São Paulo was created in 2011, information from medical records was individually reviewed and patients who met the 2001 criteria for VM, but not the new 2012 criteria, or those showing dubious information about it, were excluded.

Due to the wide variation in the literature regarding the terms used in otoneurology (dizziness, vertigo, etc.), in this article we decided to use the terms proposed for the “Classification of Vestibular Symptoms”, by the Bárány Society in 2009.^14^

Although it is an ambulatory care clinic, the vestibular migraine outpatient clinic was created in order to better understand this disease, which, even though having been described only in 1999, is currently the second most frequent diagnosis of vestibular diseases. The patients treated at the clinic are those who met the diagnostic criteria and were referred from the otoneurology clinic. Each clinic patient had a standardized questionnaire completed by the examiner at each visit. Among other things, the outpatient clinic patients are asked to answer two questions at every consultation: “Have you improved with treatment?” and “Give a score from 0 to 10 for your symptoms, where 0 is the minimum, or best possible and 10 is the maximum, or worst possible.” At each consultation, patients are treated during the normal routine of the clinic, by residents or fellows of otoneurology, supervised by a single advisor.

We included all records of patients with vestibular migraine and the following information was considered:

- Epidemiological data: name, gender, age, profession and place of birth.
- Clinical characteristics of the disease.
- Past medical history.
- Results of the treatments evaluated through the generic question “Have you improved with treatment?” and through the Visual Analog Scale (VAS).

Files of patients with another disorder causing dizziness and/or headache, those who did not meet the 2012 criteria for VM and those with illegible records or with incomplete or conflicting information were excluded.

Each patient was evaluated through the VAS for the 30 days prior to prophylactic treatment and VAS for three months after the beginning of prophylactic treatment with different drugs. The VAS was applied to the symptom headache and Vestibular Symptoms (VS). Vestibular symptoms varied somewhat among patients. Some had spontaneous vertigo, others positional vertigo, while others had vestibular-visual symptoms. Due to this variation, these symptoms were grouped as VS.

Clinical improvement was determined by the difference between these scores, called therapeutic response. In case of need for treatment change due to drug failure or side effects, or for financial reasons, the initial drug was changed to a second medication. At the VM outpatient clinic, the choice of drugs is based on the patient's profile and choice, for example, antidepressants for those with anxiety and antihypertensive agents for those with associated hypertension.

The period of three months between the interventions (definition of improvement/failure) was determined based on the fact that the therapeutic effect of drugs only begins after 2–4 weeks and the fact that the disease has natural periods of improvement and worsening and that patients, in a given month, can have more or fewer crises due to natural episodic nature of the disease and not due to real improvement/worsening. All patients receive the same advice on the benefits of eating habits, sleep hygiene, physical activity practice and identification and interruption of migraine triggers.

Pre- and post-treatment gain values were compared using the Kruskal–Wallis test. Other statistical tests used were the equal proportions test and Spearman's correlation test. Differences were considered statistically significant when *p* < 0.05 for a 95% confidence interval.

## Results

### Epidemiological results

After the analysis of 88 medical records of patients diagnosed with VM, 47 were maintained according to the inclusion and exclusion criteria. The study group had a mean age of 45.9 years (range 19–69 years) and mean duration of symptoms of 10.8 years for headache and 6.0 years for Vestibular Symptoms (VS). Due to the retrospective nature of the study, some data were not identified in the patients’ records and could not be obtained by phone contact. Because of this, some data appear with a different number from the original 47 (Table 1). There was a predominance of women (93.6%). Nearly a third of patients did not have any comorbidity and, among those with comorbidities, hypertension was the most prevalent one (Table 2).

**Table 1:** Age and duration of symptoms (years) in patients with VM.

	Age	Time headache	Time of VS
Mean	45.9	10.8	6.0
Median	47.5	7.0	4.0
Deviation	10.9	11.0	6.5
Minimum	19	1.0	0.3
Maximum	69	50	30
*n*	46	46	47

VM, vestibular migraine; VS, vestibular symptoms.

**Table 2:** Comorbidities in patients with VM.

Disease	*n*	%
None	15	31.9
SAH	15	31.9
DM	2	4.3
Dyslipidemia	9	19.1
Epilepsy	2	4.3
Hypothyroidism	6	12.8
Anxiety/depression	8	17.0
Others	14	29.8

VM, vestibular migraine; SAH, systemic arterial hypertension; DM, diabetes mellitus.

### Benefits of drug prophylaxis

Most of the patients (80.9%) reported improvement with the use of different prophylactic drugs (Table 3), with a statistically significant difference (*p* < 0.001) when compared to those who said they had not improved.

**Table 3:** Clinical improvement in patients with VM.

Improved?	Yes		*p*
	*n*	%	
Amitriptyline 25	10	76.9%	0.006
Amitriptyline 50	2	100%	0.046
Flunarizine 10	10	90.9%	<0.001
Nortriptyline 50	1	100%	a
Propranolol 40	2	66.7%	0.083
Propranolol 80	4	100%	0.005
Topiramate 100	4	57.1%	0.094
Topiramate 200	1	100%	a
Valproate 1000	1	100%	a
Valproate 500	2	66.7%	0.083
Venlafaxine 75	1	100%	a
Overall	38	80.9%	<0.001

VM, vestibular migraine.

a *p* not calculated due to small *n*.

When quantitatively evaluating the VAS values for headache and vestibular symptoms, these were lower in the post-treatment period, confirming the qualitative finding that there was clinical improvement (Table 4, Table 5). All patients showed improvement for both symptoms with a statistically significant difference (*p* < 0.001).

**Table 4:** Values obtained with the visual analog scale for headaches, before and after use of prophylactic drug, according to the drug used in patients with VM.

Prophylaxis	Headache pre		Headache post		*p*
	Mean	*n*	Mean	*n*	
Amitriptyline 25	7.54	13	2.82	11	<0.001
Amitriptyline 50	6.00	2	4.00	2	0.317
Flunarizine 10	7.73	11	3.22	9	0.002
Nortriptyline 50	8.00	1	3.00	1	0.317
Propranolol 40	6.00	3	3.00	3	0.178
Propranolol 80	8.75	4	4.00	4	0.015
Topiramate 100	8.71	7	4.33	6	0.002
Topiramate 200	10.00	1	8.00	1	0.317
Valproate 1000	7.00	1	0.00	1	0.317
Valproate 500	9.00	2	3.00	2	0.121
Venlafaxine 75	10.00	1	0.00	1	0.317
Overall	7.87	46	3.32	41	<0.001

VM, vestibular migraine.

**Table 5:** Values obtained with the visual analog scale for vestibular symptoms, before and after use of prophylactic drug, according to the drug used in patients with VM.

Prophylaxis	VS pre		VS post		*p*
	Mean	*n*	Mean	*n*	
Amitriptyline 25	6.38	13	2.55	11	0.001
Amitriptyline 50	6.50	2	2.50	2	0.221
Flunarizine 10	6.82	11	4.56	9	0.050
Nortriptyline 50	8.00	1	2.00	1	0.317
Propranolol 40	6.33	3	4.00	3	0.197
Propranolol 80	7.75	4	3.25	4	0.034
Topiramate 100	8.29	7	3.33	6	0.006
Topiramate 200	7.00	1	0.00	1	0.317
Valproate 1000	5.00	1	0.00	1	0.317
Valproate 500	7.50	2	2.00	2	0.121
Venlafaxine 75	10.00	1	0.00	1	0.317
Overall	7.04	46	3.05	41	<0.001

VM, vestibular migraine; VS, vestibular symptoms.

How much each prophylactic medication improved patients’ symptoms, calculated as the difference between pre- and post-treatment VAS (treatment response), also showed improvement (Table 6). When comparing each value of therapeutic response obtained with each type of prophylaxis used, excluding the cases of drugs used in only one or two individuals, it was observed that there was no statistically significant difference among different medications (Table 7).

**Table 6:** Therapeutic response assessed by the mean variation of VAS after prophylactic treatment in patients with VM.

Prophylaxis	Headache		VS	
	Mean	*n*	Mean	*n*
Amitriptyline 25	−4.82	11	−4.00	11
Amitriptyline 50	−2.00	2	−4.00	2
Flunarizine 10	−4.33	9	−2.56	9
Nortriptyline 50	−5.00	1	−6.00	1
Propranolol 40	−3.00	3	−2.33	3
Propranolol 80	−4.75	4	−4.50	4
Topiramate 100	−4.50	6	−5.00	6
Topiramate 200	−2.00	1	−7.00	1
Valproate 1000	−7.00	1	−5.00	1
valproate 500	−6.00	1	−6.00	1
Venlafaxine 75	−10.00	1	−10.00	1
Overall	−4.53	40	−4.10	40

VAS, visual analog scale; VM, vestibular migraine; VS, vestibular symptoms.

**Table 7:** Therapeutic response appraised by the mean variation of VAS after prophylactic treatment in patients with VM per drug used.

Prophylaxis	Headache			VS		
	Mean	*n*	*p*	Mean	*n*	*p*
Amitriptyline 25	−4.82	11	0.977	−4.00	11	0.608
Flunarizine 10	−4.33	9		−2.56	9	
Propranolol 40	−3.00	3		−2.33	3	
Propranolol 80	−4.75	4		−4.50	4	
Topiramate 100	−4.50	6		−5.00	6	
Overall	−4.53	40		−4.10	40	

VM, vestibular migraine; VS, vestibular symptoms.

### Statistical correlations for treatment strategies

We sought to assess whether there was an association between how much the patient improved (treatment response) and duration of symptoms, using Spearman's correlation test. There was no statistically significant association with any group of drugs, but regarding the overall group, there was a directly proportional and statistically significant association (*p* = 0.037) between duration of dizziness reported by the patient and the observed therapeutic response for the VS, as shown in Table 8.

**Table 8:** Statistical correlation between the response to therapy assessed by VAS after prophylactic treatment in patients with VM and time of symptoms (migraine and VS).

Prophylaxis		Time of headache		Time of VS	
		Correl. (*r*)	*p*	Correl. (*r*)	*p*
Amitriptyline 25	Pain	−10.4%	0.761	−12.3%	0.719
	SV	11.5%	0.735	48.4%	0.132
Flunarizine 10	Pain	47.0%	0.202	45.3%	0.221
	SV	−61.4%	0.079	9.3%	0.812
Propranolol 40	Pain	50.0%	0.667	100.0%	–
	SV	0.0%	1.000	86.6%	0.333
Propranolol 80	Pain	100.0%	–	100.0%	–
	SV	−100.0%	–	33.3%	0.667
Topiramate 100	Pain	77.0%	0.073	77.0%	0.073
	SV	35.3%	0.493	35.3%	0.493
Overall	Pain	23.5%	0.150	30.4%	0.056
	VS	14.8%	0.368	33.1%	0.037

VAS, visual analog scale; VM, vestibular migraine; VS, vestibular symptoms.

The group of hypertensive patients using β-blockers as prophylactic treatment for vestibular migraine and the group of anxious/depressed patients that used antidepressants, however, showed no statistically significant advantage when using these drug groups when compared to those using other drugs (Table 9, Table 10).

**Table 9:** Comparison between therapeutic response measured by VAS variation in hypertensive patients receiving β-blocker drug prophylaxis and hypertensive patients receiving other drugs.

Drug	Response for pain		Response for VS	
	Others	Propranolol 40	Others	Propranolol 40
Mean	−4.20	−3.50	−4.60	−3.50
*n*	10	2	10	2
*p*	0.914		0.746	

VAS, visual analog scale; VS, vestibular symptoms.

**Table 10:** Comparison of therapeutic response at VAS for anxious individuals treated with antidepressant prophylaxis and VAS for anxious individuals treated with other drugs.

Anxiety	Response for pain		Response for VS	
	Others	Antidepressants	Others	Antidepressants
Mean	−3.67	−10.00	−4.17	−5.00
*n*	6	1	6	1
*p*	0.094		1.000	

VAS, visual analog scale; VS, vestibular symptoms.

There was no statistically significant difference among treatment responses obtained with the three different treatment regimens used: one drug, one medication after substitution due to treatment failure or the combination of two drugs (Table 11).

**Table 11:** Comparison of the therapeutic responses measured by VAS among the three treatment regimens used: a medication, a substitute medication after the failure of the first one and association of two drugs.

	Regimen	Mean	*n*	*p*
Therapeutic response for headache	One medication	−4.53	41	0.174
	A substitute medication	−3.50	15	
	Association of two medications	−2.50	7	

Therapeutic response for VS	One medication	−4.10	41	0.093
	A substitute medication	−2.21	15	
	Association of two medications	−4.67	7	

VAS, visual analog scale; VS, vestibular symptoms.

## Discussion

Vestibular migraine shows a predominance of female patients, with a ratio of 1.5–5:1, occurring at any age and the headache usually precedes vestibular symptoms in most patients.^7^, ^15^ The same was observed in this sample with. 93.6% of women, mean age of 45.9 years and mean duration of headache longer than the mean time of vestibular symptoms (Table 1).

The benefit of drug prophylaxis for VM is still a vast field of study. Overall, it is accepted that the prophylactic treatment for VM is effective, but there are no double-blind randomized trials comparing different treatments.^12^, ^13^ Due to the recent description of VM, only studies on migraine associated with dizziness or imbalance were found in the literature. Reploeg and Goebel reported 72% of patients with vertigo and imbalance symptoms improved after prophylactic treatment.^16^ Similar results were observed in this study, in which 80.9% of patients reported some improvement with the use of different prophylactic drugs (Table 3).

No studies were found comparing groups of prophylactic drugs in patients with diagnostic criteria for VM. Even review articles on this topic failed to reach conclusions about the best drug choice for prophylaxis.^12^, ^13^, ^17^

The therapeutic response to episodic disorders, such as Meniere's disease or vestibular migraine is more difficult to study due to natural disease fluctuations. Disease improvement after one month, for instance, may be due to a natural asymptomatic period, rather than due to a therapeutic effect of an intervention. The longer the period of evaluation of therapeutic response, the more reliable it will be. The 3-month period used in this study was empirically chosen, respecting the minimum of 2–4 weeks for drug action onset and adding 2 more months. Duration longer than the 3-month period could lead to lower adherence to treatment by patients.

This study also showed quantitative benefits with drug treatment for VM prophylaxis, when using the VAS. Despite the imperfections of this assessment method, some things may be postulated. There was a statistically significant improvement in headache and VS, but some drugs alone showed improvement with no significant difference. Daily doses of 25 mg of amitriptyline; 10 mg of flunarizine; 80 mg of propranolol and 100 mg of topiramate showed symptom improvement with statistically significant difference. It is noteworthy that this occurred also for both the symptom of headache and for the VS, and, likewise, the drugs that showed no statistical difference for vestibular symptom improvement were the same that showed no improvement for the symptom of headache.

Improvement without statistical significance was observed for amitriptyline 50 mg and topiramate 200 mg; although smaller doses have shown a significant improvement, that must be due to the fact that these doses were used precisely in patients who did not respond to lower doses, that is, these were patients with refractory symptoms, despite the small sample size. As for nortriptyline 50 mg; valproate 500–1000 mg, and venlafaxine75 mg, the low number of patients in the sample may have been responsible for the nonsignificant improvement. Finally, propranolol seems to require a higher dose (80 mg/day) to show improvement.

The improvement in VAS values was similar among the different drugs used (Table 7). This can be seen as a positive result, as all drugs led to an improvement subjectively perceived by the patient, both for the headache symptom and the VS. It remains, however, difficult to choose the best option for different patients with VM and the choice is made according to possible drug contraindications or interactions with other medications that patients are already using, trying to optimize patient drug therapy.

According to a study by Bikhasi et al.,^18^ the temporal association between dizziness and migraine did not influence the efficiency of prophylactic treatment. In this study, however, a statistically significant association between duration of dizziness and therapeutic response for dizziness was observed (*p* = 0.037). The duration of dizziness also showed a tendency toward a negative association for therapeutic response for headache (*p* = 0.056). This shows that the longer the duration of VS experienced by the patient, the better the therapeutic response provided by prophylactic drug therapy. Specific studies on this topic are needed to better answer the question “Does the duration of vestibular symptoms influence the effectiveness of drug prophylaxis?”

The choice of prophylactic drug is based on the patient's profile or comorbidities (antidepressants for patients diagnosed with anxiety, for instance), with no particular advantage of one group of drugs over the others.^11^, ^12^, ^13^, ^17^ In the current sample, the group of hypertensive patients who used beta-blockers as prophylaxis for VM and the group of anxious/depressed patients who used antidepressants did not show any statistically significant advantage with the use of these drug groups compared to those who used other drugs. This fact does not invalidate the current recommendations for the choice of prophylactic drugs; however, it raises the suspicion (given the small sample size) that there is no particular advantage in improving VM symptoms. Naturally, in the context of rational drug use, it is more appropriate to use a single drug that is effective for two patient comorbidities than the use of one drug for each disease.

Although the group of patients with substitution drug and association of drugs to the original regimen was small, that prevented a more detailed study, the absence of statistical difference among the groups of patients who required the substitution of the prophylactic drug supports the current recommendations that there is no group of drugs better than the other, in this case, even when two drugs were used in combination.

## Conclusions

The prophylactic medications used to treat VM seem to improve the symptoms of this disease. There is no group of prophylactic drugs exhibiting better results than others. The longer the duration of VS experienced by the patient, the better is the therapeutic response to the prophylactic drugs used. There is no further advantage, in addition to drug therapy optimization, related to the reduction in headaches and VS when using antidepressants in patients with a diagnosis of depression or antihypertensive medications in patients with SAH.

## Conflicts of interest

The authors declare no conflicts of interest.
